# Cervical Stiffness Measured by the Cervisense Intravaginal Probe for Identification of Imminent Delivery in Symptomatic Threatened Preterm Labor

**DOI:** 10.3390/medicina62050830

**Published:** 2026-04-27

**Authors:** Andrea Samper-Girona, Amaia Aiartzaguena, Jorge Burgos, María D. Muñoz-González, María M. Gil, Amelia Valladolid, Alain Urones, Teresa Cobo, Silvia Ferrero, María Goya, Ester Del Barco Martínez, José E. Blanco-Carnero, Pilar Prats Rodríguez, Francisca S. Molina

**Affiliations:** 1 Hospital Universitario Clínico San Cecilio, 18016 Granada, Spain; asampergirona@gmail.com; 2 Instituto de Investigación Biosanitaria ibs.GRANADA, 18012 Granada, Spain; 3 Hospital Universitario Cruces, 48903 Barakaldo, Spain; aiartzaguena.amaia@gmail.com (A.A.); jorge.burgossancristobal@osakidetza.eus (J.B.); 4 Hospital Universitario de Torrejón, 28850 Madrid, Spain; mdmunoz.ginecologia@gmail.com (M.D.M.-G.); mar.gilmira@bcm.edu (M.M.G.); 5 School of Medicine, Universidad Francisco de Vitoria, 28223 Madrid, Spain; 6 Hospital Universitario Basurto, 48013 Bilbao, Spain; amelia.valladolidurdangaray@osakidetza.eus (A.V.); alain.uronesgoikoetxea@osakidetza.eus (A.U.); 7 Hospital Clínic de Barcelona, 08036 Barcelona, Spain; tcobo@clinic.cat (T.C.); silviairene.ferrero@sjd.es (S.F.); 8 Hospital Sant Joan de Déu, 08950 Barcelona, Spain; 9 Hospital Universitario Vall d`Hebron, 08035 Barcelona, Spain; maria.goya@vallhebron.cat (M.G.); ester.delbarco@vallhebron.cat (E.D.B.M.); 10 School of Medicine, Universitat Autònoma de Barcelona, 08193 Barcelona, Spain; 11 Hospital Clínico Universitario Virgen de la Arrixaca, 30120 Murcia, Spain; jeblancoc@gmail.com; 12 Hospital Universitari Dexeus, 08028 Barcelona, Spain; pilpra@dexeus.com

**Keywords:** cervical stiffness, threatened preterm labor, torsional wave elastography, transvaginal cervical length, short-term prediction

## Abstract

*Background and Objectives*: Risk stratification in symptomatic threatened preterm labor (TPTL) remains challenging, particularly for clinically actionable time horizons. We evaluated whether quantitative cervical stiffness measured with the Cervisense Intravaginal Probe discriminates between women who will and will not deliver within 7, 10, or 14 days after presentation and assessed its discriminative performance alone and in combination with transvaginal cervical length. *Materials and Methods*: We conducted a multicenter, non-interventional study across 10 obstetric emergency units in Spain. Eligible participants were women aged ≥ 18 years with a live singleton pregnancy at 28^+0^ to 36^+6^ weeks, regular uterine contractions, intact membranes, and cervical dilatation < 2 cm on speculum examination. Cervical stiffness was measured at presentation using torsional wave elastography with the Cervisense Intravaginal Probe. Cervical length was measured by transvaginal ultrasonography following international standards. Outcomes were delivery within 7, 10, and 14 days after presentation. Discrimination was quantified using the area under the receiver operating characteristic curve (AUC). An explanatory multivariable logistic regression model for delivery within 14 days included cervical stiffness, cervical length, and covariates selected as potential confounders. *Results*: Among 305 participants, 17 (5.6%) delivered within 7 days, 19 (6.2%) within 10 days, and 24 (7.9%) within 14 days. Mean cervical stiffness was lower among women who delivered within 14 days than among those who did not (8.63 vs. 14.82 kPa; *p* = 0.048). Cervical length was shorter among women who delivered within 7, 10, and 14 days (all *p* < 0.001). Discrimination for cervical stiffness was moderate (AUC, 0.66, 0.64, and 0.73 for ≤7, ≤10, and ≤14 days) and higher for cervical length (AUC, 0.80, 0.77, and 0.78). The combined model achieved the highest discrimination (AUC, 0.85, 0.85, and 0.86). In the ≤14-day explanatory model, higher log-transformed cervical stiffness was associated with lower odds of delivery (OR, 0.907; 95% CI, 0.828 to 0.995; *p* = 0.039), as was longer cervical length (OR per 1 mm, 0.910; 95% CI, 0.876 to 0.946; *p* < 0.001). *Conclusions*: In symptomatic TPTL, Cervisense-derived cervical stiffness showed moderate discrimination for short-term delivery and provided complementary information to cervical length, with improved discrimination when both measures were combined. These findings support future studies developing multivariable prediction models that incorporate quantitative cervical consistency.

## 1. Introduction

Preterm labor (PTL) is a major driver of neonatal morbidity and mortality. Complications of prematurity are the most common cause of death among children younger than 5 years [1]. Survivors are at increased risk of long-term comorbidity, adverse neurodevelopmental outcomes, and social disability [2,3]. In this context, symptomatic threatened preterm labor (TPTL) represents a high-stakes presentation that prompts urgent obstetric assessment because management decisions depend on the probability of imminent delivery [4]. However, symptoms have limited specificity, and fewer than 10% of patients presenting with TPTL deliver within 7 days [5,6]. Consequently, imprecise short-term risk stratification can expose many patients to unnecessary interventions and avoidable resource use while delaying escalation of care for those who will deliver soon.

Current risk assessment in symptomatic TPTL relies on clinical examination complemented by tests that record uterine activity and cervical length (CL) or biochemical tests associated with higher risk of delivery. Digital examination is limited by subjectivity and interobserver variability [7], and transvaginal ultrasonography of CL, although widely used, remains an imperfect predictor of imminent delivery. In symptomatic patients, a cervical-length cut-off between 10 and 20 mm identifies only approximately 30 to 45% of deliveries within 7 days [5,8], and measurement accuracy can be affected by operator experience, uterine contractions at the time of assessment, fetal presentation, maternal obesity, and many other factors that can induce measurement error [9]. Biochemical tests based on cervicovaginal biomarkers, including fetal fibronectin (fFN) and placental alpha microglobulin-1 (PAMG-1), have also been proposed to refine short-term risk stratification and, in some studies, have achieved sensitivities above 70% and specificities above 90% [10]; however, the evidence is highly heterogeneous, likely reflecting variation in sampling conditions and baseline risk across populations, which limits transportability and consistent clinical implementation.

Therefore, current diagnostic tools, although clinically informative, remain insufficient to reliably discriminate true from false TPTL, which limits their utility for guiding time-sensitive management decisions. This gap underscores the need for objective, reproducible, and quantitative measures of cervical remodeling that can complement existing approaches and improve identification of patients at genuine risk of imminent preterm delivery. Experimental and clinical evidence indicates that cervical softening driven by extracellular matrix remodeling precedes measurable cervical shortening, suggesting that cervical biomechanical properties may provide earlier risk signals than anatomical assessment alone [11,12]. On this physiological basis, we developed the Cervisense Intravaginal Probe to quantify cervical stiffness using torsional wave elastography, which measures propagation of low-energy torsional waves through cervical tissue to estimate the shear modulus [13,14,15].

In this study, we evaluated the discriminative performance of cervical stiffness measured with the Cervisense Intravaginal Probe at presentation among patients with symptomatic TPTL to identify those who deliver within 7, 10, or 14 days, and assessed whether cervical stiffness provides complementary information to transvaginal CL.

## 2. Materials and Methods

### 2.1. Study Design and Setting

This multicenter and non-interventional study was conducted in obstetric emergency units at 10 hospitals across Spain from 2021 to 2024. All sites adhered to a standardized operating procedure and received on-site training before starting. A total of 305 pregnant women were enrolled. Enrollment by center was as follows: in Biscay, Hospital Universitario de Basurto with 42 participants (13.8%) and Hospital Universitario de Cruces with 58 (19.0%); in Barcelona, Hospital Universitario Dexeus with 23 (7.5%), Hospital Clínic de Barcelona with 22 (7.2%), Hospital Sant Joan de Déu with 10 (3.3%), and Hospital Universitari Vall d’Hebron with 13 (4.3%); in Granada, Hospital Universitario Clínico San Cecilio with 56 (18.4%); in Murcia, Hospital Universitario Virgen de la Arrixaca with 25 (8.2%); and in Madrid, Hospital Quirónsalud San José with 3 (1.0%) and Hospital Universitario de Torrejón with 53 (17.4%).

### 2.2. Participants

Eligible participants were pregnant women aged 18 years or older with a live singleton fetus between 28 weeks and 0 days and 36 weeks and 6 days of gestation, regular uterine contractions at presentation, intact amniotic membranes, cervical dilation less than 2 cm on speculum examination, and written informed consent for study participation. Women were excluded if they had a latex allergy, prolapsed membranes with an hourglass appearance, major fetal malformation, suspected chorioamnionitis, active vaginal bleeding, a cervical cerclage in place, Müllerian anomalies, or a pessary in use.

The sample size was prespecified using the Riley framework for prediction model development [16]. Three criteria were applied: precise estimation of the intercept assuming an expected event proportion of 0.15, limitation of the mean absolute prediction error to 0.045 for a model with up to seven parameters, and achievement of a global shrinkage factor of at least 0.90 under an anticipated C-statistic of 0.85. The largest requirement across criteria was selected, yielding a target development sample of 289 participants, corresponding to approximately 43 events and about 6 events per parameter.

### 2.3. Study Procedures and Variables

Women presenting with symptoms of TPTL were screened at the obstetric emergency visit. Those with more than eight uterine contractions during a 60 min observation period were invited to participate. After provision of written study information and informed consent, participants underwent a speculum examination. A cervicovaginal sample was then collected for fFN o PAMG-1 testing, followed by cervical stiffness assessment during the same visit using the Cervisense Intravaginal Probe (Ultrasound-Innovation Medtech SL, Bizkaia, Spain) while the speculum remained in place. After removal of the speculum, CL was measured by transvaginal ultrasonography in accordance with international standards [17,18]. Digital vaginal examination and Bishop scoring were performed at the discretion of the treating clinician. Subsequent management, including administration of tocolytics, antenatal corticosteroids, and antibiotic prophylaxis for group B streptococcus, followed local clinical practice at each participating center.

Participants were followed after the index visit to ascertain delivery status within prespecified horizons of 7, 10, and 14 days, classifying cases with delivery within the corresponding time window as true TPTL and those without delivery as false TPTL for that horizon. The 10-day and 14-day horizons were included to explore whether the discriminative performance of cervical stiffness extends beyond the 7-day window, given that clinical decisions regarding hospital admission, transfer, and repeat corticosteroid courses may be relevant within a broader timeframe.

Variables collected comprised sociodemographic characteristics (age, ethnicity, and date of admission or consultation for TPTL) and clinical history (prepregnancy weight, current weight, height, smoking status, relevant comorbidities with timing of diagnosis, concomitant medication and dietary supplements with indication and timing, and prior reproductive tract interventions with timing). Pregnancy-related variables included gestational age at presentation, mode of conception (spontaneous or assisted reproductive technology), designation of high-risk pregnancy, gravidity, parity with gestational age of prior births, prior pregnancy losses with gestational age, and prior episodes of threatened preterm labor in the current or previous pregnancies with dates and counts. Transvaginal ultrasonographic cervical-length measurements were performed in accordance with international standards [17,18]. Symptom and presentation variables included vaginal bleeding or abdominal cramping with or without diarrhea, contractions associated with lumbar pain or suprapubic pressure, vaginal discharge or fluid loss with recorded characteristics, spotting characteristics, contraction frequency and type, and whether hospital admission occurred, together with treatments administered. Neonatal and delivery variables included birth weight, gestational age at delivery, neonatal length, perinatal death, delivery-room resuscitation, Apgar scores, date of delivery, mode of delivery, and onset of labor (spontaneous, induced, or without labor).

#### Cervical Stiffness Measurement

Cervical stiffness was measured at the index visit using the Cervisense Intravaginal Probe following a standardized acquisition protocol. A single-use device cover was applied to the probe, and a vacuum activation mechanism was used to secure full adhesion of the cover to the sensor surface; the operator verified complete adherence without air pockets to minimize measurement artifacts. The integrated microcamera was connected to a laptop via USB and visualized using the dedicated Cervisense camera application. An initial calibration step was performed outside the patient to capture ambient signals. After placement of a vaginal speculum, the probe was introduced into the vaginal canal and it was then advanced until the sensor was in direct contact with the anterior cervical lip. Operators followed real-time software guidance to confirm correct positioning and adequate contact pressure. Upon activation, the device generated a low-energy torsional wave and recorded the tissue response using torsional wave elastography, from which cervical stiffness was estimated as the shear modulus and reported by the software as the Cervisense stiffness value (kPa).

The device performs three sequential stiffness measurements at each assessment, from which a single stiffness value is reported. In the present cohort, the internal consistency of these three measurements was good, with an intraclass correlation coefficient for the average of measurements on the absolute-agreement model of 0.83 (95% CI, 0.78 to 0.87; *p* < 0.001). Reproducibility and technical performance of the device have been characterized in detail elsewhere [14,15].

To evaluate device safety, all adverse events occurring within 7 days after the measurement were prospectively recorded. Each event was reviewed by the research team, who classified causality into prespecified categories: not related, possible, probable, or definitely related to the device.

### 2.4. Statistical Analysis

Maternal baseline characteristics were summarized using medians and interquartile ranges for continuous variables and counts with percentages for categorical variables. Between-group comparisons for continuous variables were performed using the Wilcoxon rank-sum test, and categorical variables were compared using Pearson’s chi-squared test or Fisher’s exact test, as appropriate.

Cervical stiffness (shear modulus, kPa) was approximately log-normally distributed. For each horizon, differences in cervical stiffness were estimated using log-normal regression with cervical stiffness as the dependent variable and delivery within the horizon as a binary predictor. Heteroscedasticity-consistent (HC3) robust standard errors were used to derive 95% confidence intervals and two-sided *p* values. Cervical length (mm) was analyzed on the original scale, with mean differences between outcome groups estimated using linear regression with robust standard errors. These univariable comparisons were performed on complete cases for the corresponding variable.

Discrimination was quantified for cervical stiffness alone, cervical length alone, and their combination using receiver operating characteristic curves and the area under the curve (AUC) with 95% confidence intervals for each time horizon. For the exploratory multivariable analysis of delivery within 14 days, a logistic regression model was fitted including cervical length and cervical stiffness together with covariates identified as potential confounders. Covariate selection was based on three criteria: clinical relevance to spontaneous preterm delivery, univariable association with the outcome, and plausible association with cervical stiffness that could confound the stiffness–outcome relationship. Data-driven variable selection procedures were not applied. Cervical stiffness was incorporated using restricted cubic splines to allow for nonlinearity. Missing data in the multivariable model were handled using multiple imputation by chained equations with 50 imputed datasets; the imputation model included all variables specified in the analysis model, and parameter estimates were pooled across imputed datasets using Rubin’s rules. Effects were summarized as odds ratios with 95% confidence intervals, and for cervical stiffness, the reported estimate corresponded to the average marginal effect across the observed distribution of stiffness values. All statistical analyses were performed using R software, version 4.5.2 [19].

### 2.5. Ethical Issues

The study was approved by the Euskadi Research Ethics Committee (reference PS2020048). Clinical study authorization was granted by the Spanish Agency for Medi-cines and Health Products (reference 854/20/EC), and the study was conducted in accordance with international Good Clinical Practice (GCP) guidelines, the Declaration of Helsinki in its latest version, and applicable international and national rules and regulations.

## 3. Results

### 3.1. Participant Characteristics

Among 305 women presenting with symptomatic threatened preterm labor, 17 (5.6%) delivered within 7 days of the index visit, 19 (6.2%) within 10 days, and 24 (7.9%) within 14 days. Overall, participants had a median age of 32.0 years (interquartile range, 28.0 to 35.0) and presented at a median gestational age of 32.0 weeks (30.0 to 34.0); most were Caucasian (96.4%) and pregnancies were predominantly conceived spontaneously (92.1%) (Table 1). Baseline maternal characteristics were broadly similar between women who delivered within 14 days and those who did not, although cigarette smoking was more frequent in the ≤14-day group (29.2% vs. 9.6%; *p* = 0.010) and gestational age at presentation was slightly higher (median, 33.0 vs. 32.0 weeks; *p* = 0.011) (Table 1).

### 3.2. Cervical Stiffness and Cervical Length by Short-Term Delivery Status

Of 305 participants, 242 (79.3%) had valid cervical stiffness measurements. Among the 63 participants with invalid measurements, one delivered within 7 days of presentation. Cervical stiffness was lower among women who delivered within 7, 10, and 14 days than among those who did not, although the between-group differences were imprecisely estimated for the 7-day and 10-day horizons. For delivery within 7 days, mean cervical stiffness was 8.50 kPa (95% CI, 3.84 to 18.82) among women who delivered and 14.59 kPa (95% CI, 12.54 to 16.99) among those who did not (mean difference [MD], −6.10 kPa; 95% CI, −13.21 to 1.02; *p* = 0.093). For delivery within 10 days, mean stiffness was 9.41 kPa (95% CI, 4.53 to 19.57) versus 14.55 kPa (95% CI, 12.48 to 16.95) (MD, −5.14 kPa; 95% CI, −12.37 to 2.10; *p* = 0.164). For delivery within 14 days, stiffness was 8.63 kPa (95% CI, 4.45 to 16.74) versus 14.82 kPa (95% CI, 12.74 to 17.25) (MD, −6.19 kPa; 95% CI, −12.33 to −0.05; *p* = 0.048).

**Figure 1:**
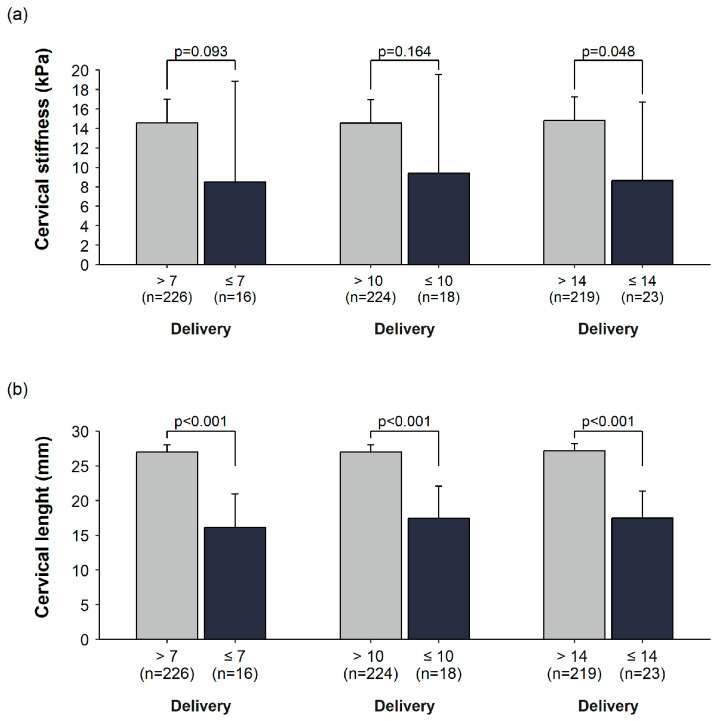
Cervical stiffness (**a**) and cervical length (**b**) by delivery within 7, 10 and 14 days after presentation.

Cervical length differed consistently across all time horizons. Mean cervical length was shorter among women who delivered within 7 days than among those who did not (16.12 mm [95% CI, 11.29 to 20.96] vs. 27.01 mm [95% CI, 26.00 to 28.01]; MD, −10.88 mm; 95% CI, −15.83 to −5.94; *p* < 0.001). Similar differences were observed for delivery within 10 days (17.44 mm [95% CI, 12.79 to 22.10] vs. 27.00 mm [95% CI, 25.99 to 28.01]; MD, −9.56 mm; 95% CI, −14.32 to −4.79; *p* < 0.001) and within 14 days (17.48 mm [95% CI, 13.61 to 21.34] vs. 27.17 mm [95% CI, 26.15 to 28.18]; MD, −9.69 mm; 95% CI, −13.69 to −5.69; *p* < 0.001). These distributions are shown in Figure 1.

**Figure 2:**
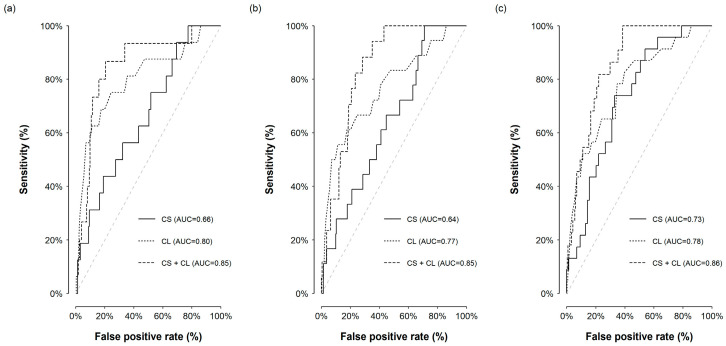
Receiver operating characteristic curves for cervical stiffness and cervical length. Panel (**a**) shows discrimination for delivery within 7 days, panel (**b**) for delivery within 10 days, and panel (**c**) for delivery within 14 days after presentation. Curves are shown for cervical stiffness (CS), cervical length (CL), and the combined model (CS + CL).

Cervical stiffness showed moderate discrimination for short-term delivery, with AUCs of 0.66 (95% CI, 0.53 to 0.80) for delivery within 7 days, 0.64 (95% CI, 0.52 to 0.77) within 10 days, and 0.73 (95% CI, 0.63 to 0.82) within 14 days. Cervical length had higher discrimination across all horizons, with AUCs of 0.80 (95% CI, 0.67 to 0.93), 0.77 (95% CI, 0.64 to 0.89), and 0.78 (95% CI, 0.67 to 0.88) for delivery within 7, 10, and 14 days, respectively. Combining cervical stiffness and cervical length further improved discrimination, yielding AUCs of 0.85 (95% CI, 0.74 to 0.95) for delivery within 7 days, 0.85 (95% CI, 0.78 to 0.91) within 10 days, and 0.86 (95% CI, 0.80 to 0.92) within 14 days (Figure 2).

### 3.3. Multivariable Logistic Regression for Delivery Within 14 Days

In the multivariable model for delivery within 14 days, both cervical stiffness and cervical length retained independent associations with the outcome (Table 2). Higher log-transformed cervical stiffness was associated with lower odds of delivery within 14 days (OR, 0.907; 95% CI, 0.828 to 0.995; *p* = 0.039), which corresponds to an approximately 9% reduction in the odds of delivery for each 1-unit increase in log stiffness. Longer cervical length was also associated with lower odds of delivery (OR per 1 mm increase, 0.910; 95% CI, 0.876 to 0.946; *p* < 0.001). Cigarette smoking was associated with higher odds of delivery within 14 days (OR, 4.378; 95% CI, 2.231 to 8.589; *p* < 0.001). The remaining covariates showed no statistically significant associations in this model and are reported in Table 2.

### 3.4. Device Safety

Within 7 days after measurement, 22 adverse events were recorded (Table 3). Most events were classified as not related to the device, and events with any degree of suspected relatedness were limited to genital bleeding. Cervical bleeding was classified as not related in 1 event (14.3%), possible in 4 (57.1%), and probable in 2 (28.6%). Vaginal bleeding was classified as possible in 1 event (33.3%), probable in 1 (33.3%), and definitely related in 1 (33.3%). All bleeding episodes were classified as mild.

## 4. Discussion

In this multicenter cohort of women presenting with TPTL, cervical stiffness quantified with the Cervisense Intravaginal Probe was lower among those who delivered within 7, 10, and 14 days after presentation, with the between-group difference reaching statistical significance for the 14-day horizon. Discrimination of cervical stiffness alone was moderate across horizons (AUC, 0.64 to 0.73), whereas cervical length showed higher discrimination (AUC, 0.77 to 0.80). The combined model including cervical stiffness and cervical length achieved the highest discrimination for short-term delivery (AUC, 0.85 to 0.86 across horizons). In the explanatory multivariable logistic regression for delivery within 14 days, cervical stiffness and cervical length remained independently associated with the outcome, indicating complementary prognostic information beyond cervical length alone.

Across prior studies, cervical softening assessed with quantitative devices has been consistently associated with a higher likelihood of preterm delivery, supporting the biological premise that biomechanical remodeling provides prognostic information beyond symptoms alone. In symptomatic TPTL, the STIPP cohort using an aspiration-based cervical stiffness index reported lower stiffness among women delivering within 7 days and moderate discrimination for that horizon (AUC 0.77), with no correlation between cervical length and stiffness, suggesting complementary information [20]. A second prospective pilot study, also using the aspiration-based stiffness index, reported strong discrimination for delivery within 14 days and additional performance gains when stiffness and cervical length were considered jointly [21]. Evidence from ultrasound elastography similarly supports an association between reduced cervical stiffness and subsequent spontaneous preterm birth, reinforcing the concept that mechanical properties capture clinically relevant remodeling [22,23]. However, the evidence remains limited by small numbers of short-horizon events, modest sample sizes, and incomplete measurements or follow-up. Definitions of TPTL and time-to-delivery endpoints are heterogeneous, and handling of medically indicated deliveries is inconsistent. Measurement protocols and reported stiffness metrics vary across technologies and operators, and external validation with calibration and clinical utility analyses is infrequent.

Several multivariable risk prediction tools have been developed for symptomatic TPTL, including the QUIDS model and the QUantitative Innovation in Predicting Preterm birth (QUiPP) App [24,25]. The QUIDS model, based on quantitative fetal fibronectin and clinical predictors, showed high discrimination for spontaneous delivery within 7 days (AUC, 0.89), with similar performance on external validation in a large prospective UK cohort (AUC, 0.89) [24]. The QUiPP App v.2 has also undergone external validation across three European cohorts of symptomatic women, with AUCs for spontaneous delivery within 1 week after testing ranging from 0.74 to 0.84 for the combined cervical-length plus quantitative fibronectin model, and 0.80 for the cervical-length-only model in a cohort without fibronectin testing [26]. However, strong predictive performance does not necessarily translate into improved patient outcomes after implementation, and pragmatic evidence from implementation research shows that a QUiPP-guided management algorithm did not reduce unnecessary management compared with conventional care [27].

Incorporating quantitative cervical consistency into the assessment of symptomatic TPTL may be particularly valuable because it is anchored to the staged physiology of cervical remodeling. Cervical ripening is a progressive process in which early softening, driven by extracellular matrix and collagen remodeling, precedes the clinically apparent phases of effacement and dilation and can occur independently of uterine contractions [11]. Transvaginal cervical length primarily quantifies geometric shortening, which becomes detectable after substantial microstructural change has already occurred, whereas tissue softness increases progressively throughout gestation and represents a final common pathway across heterogeneous etiologies of spontaneous preterm birth [28]. A quantitative measure of cervical consistency could therefore complement cervical length by reflecting the underlying remodeling stage, potentially distinguishing women with symptoms but limited cervical softening from those with advanced tissue remodeling at higher short-term risk. From a clinical perspective, this measurement could be integrated at the point of triage in obstetric emergency units, as it is performed during the speculum examination already required for clinical evaluation, does not require additional patient preparation, and provides a quantitative result at the bedside. In combination with cervical length, it could contribute to more informed decisions regarding hospital admission, initiation or withholding of tocolysis, and administration of antenatal corticosteroids.

From an implementation perspective, the Cervisense Intravaginal Probe provides a quantitative stiffness value and incorporates acquisition guidance features, including pressure sensing, probe-placement assessment, a lateral micro-camera, and on-screen feedback, which are intended to standardize the measurement process and may reduce operator dependency relative to image-based techniques. Training is based on a structured on-site session covering probe positioning, pressure control, acquisition quality, cleaning, and data transfer, with refreshers reserved for identified deviations. Prior work reported substantial gains in reproducibility after incorporation of the lateral micro-camera and targeted operator training [14], consistent with a relatively short learning curve and a limited site-level training burden. Formal cost-effectiveness assessment is contingent on a validated prediction model and is planned for future studies.

Safety findings support feasibility for use in emergency settings. Adverse events adjudicated as possibly, probably, or definitely related to the device were limited to genital bleeding within 7 days after measurement. These data, together with the standardized measurement protocol across centers, support further evaluation in studies designed to establish externally validated prediction models with calibration and clinical utility assessment and to test stiffness-guided management algorithms in pragmatic trials with patient-centered outcomes and resource-use endpoints.

The study findings should be interpreted in light of several limitations. First, event counts were low for all horizons and particularly for the shortest (17 events for delivery within 7 days, 19 for 10 days, and 24 for 14 days), which reduced precision for between-group differences and discrimination estimates, limited the number of covariates that could be included in the multivariable model, and precludes reliable assessment of model stability. This constraint underscores that the present findings should be regarded as hypothesis-generating and require confirmation in larger cohorts. Second, clinical management after the index visit followed local protocols at each center and was not standardized across sites. Variation in the use of tocolysis, antenatal corticosteroids, and hospital admission could have influenced the interval from presentation to delivery, although this pragmatic design reflects the conditions under which the device would be used in practice. In addition, cervical length measurements were available to treating clinicians and may have influenced management decisions, which could attenuate the observed association between cervical length and short-term delivery through a treatment paradox. However, the magnitude of this effect is likely limited, as the certainty of evidence supporting a clinically meaningful delay in delivery with tocolytic agents beyond 48 h remains low [29], and cervical stiffness results were not disclosed to clinicians and therefore were not subject to this potential confounding pathway. Third, eligibility was restricted to singleton pregnancies at 28+0 to 36+6 weeks with intact membranes and cervical dilatation less than 2 cm, which may limit generalizability to other symptomatic populations. Fourth, although cervicovaginal biomarker testing (fFN or PAMG-1) was performed at all participating centers, these results were not included in the present analysis because the study was designed to evaluate the discriminative value of cervical stiffness and its combination with cervical length. Comparison with biomarker-based prediction strategies and assessment of the incremental value of cervical stiffness over existing biomarkers are planned for subsequent analyses within a formal prediction model development framework. Fifth, the multivariable logistic regression was undertaken for explanatory purposes to characterize the association between cervical stiffness and delivery within 14 days, rather than to develop a clinical prediction model, and calibration and clinical utility were therefore not evaluated. Future research should focus on developing and externally validating prediction models that incorporate cervical stiffness, with assessment of calibration and decision-analytic utility and pragmatic evaluation of stiffness-informed management strategies using patient-centered outcomes and resource-use endpoints.

## 5. Conclusions

In this multicenter cohort of women presenting with symptomatic TPTL, cervical stiffness measured with the Cervisense Intravaginal Probe was lower among those who delivered within 7, 10, and 14 days after presentation, with the clearest separation observed for the 14-day horizon. Cervical stiffness alone showed moderate discrimination for short-term delivery, whereas cervical length showed higher discrimination. Combining cervical stiffness with cervical length yielded the highest discrimination across all horizons, supporting the potential complementarity of biomechanical and anatomic assessment in short-term risk stratification. Device-related adverse events were uncommon and limited to mild genital bleeding. These findings support future research aimed at developing and externally validating multivariable prediction models that incorporate quantitative measures of cervical biomechanical properties to improve short-term risk stratification in symptomatic threatened preterm labor.

## Figures and Tables

**Table 1:** Maternal baseline characteristics by delivery within 14 days.

Characteristic	Overall N = 305 ^1^	Delivery ≤ 14 Days N = 24 ^1^	Delivery > 14 Days N = 281 ^1^	*p*-Value ^2^
*Maternal-related characteristics*				
Maternal age, years	32.0 (28.0, 35.0)	31.5 (28.0, 35.0)	32.0 (28.0, 35.0)	0.989
Maternal weight, kg	67.0 (60.8, 75.0)	68.9 (61.5, 75.5)	67.0 (60.5, 75.0)	0.742
Maternal height, cm	162.0 (157.0, 166.0)	160.0 (158.5, 165.0)	162.0 (157.0, 166.0)	0.572
Ethnicity				0.600
Caucasian	294 (96.4%)	23 (95.8%)	271 (96.4%)	
African	8 (2.6%)	1 (4.2%)	7 (2.5%)	
South Asian	3 (1.0%)	0 (0.0%)	3 (1.1%)	
Cigarette smoker	34 (11.1%)	7 (29.2%)	27 (9.6%)	0.010
Gestational age at first visit, weeks	32.0 (30.0, 34.0)	33.0 (32.0, 35.0)	32.0 (30.0, 34.0)	0.011
Conception				0.107
Natural	281 (92.1%)	20 (83.3%)	261 (92.9%)	
ART	24 (7.9%)	4 (16.7%)	20 (7.1%)	
Parity				0.259
Nulliparous	157 (51.5%)	11 (45.8%)	146 (52.0%)	
Parous preterm delivery < 30 weeks	4 (1.3%)	1 (4.2%)	3 (1.1%)	
Parous preterm delivery 30–36 weeks	22 (7.2%)	3 (12.5%)	19 (6.8%)	
Parous term delivery ≥ 37 weeks	122 (40.0%)	9 (37.5%)	113 (40.2%)	
Previous TPTL episodes	42 (13.8%)	5 (20.8%)	37 (13.2%)	0.349
Previous cervical surgery	75 (24.6%)	6 (25.0%)	69 (24.6%)	0.961
Tocolysis	71 (23.3%)	7 (29.2%)	64 (22.8%)	0.477
*Newborn-related characteristics*				
Gestational age at delivery, weeks	38.0 (37.0, 40.0)	35.0 (32.5, 36.0)	39.0 (37.0, 40.0)	<0.001
Birthweight, kg	3.2 (2.8, 3.5)	2.5 (2.1, 2.9)	3.2 (2.9, 3.5)	<0.001

^1^ Median (Q1, Q3); n (%). ^2^ Wilcoxon rank sum test; Fisher’s exact test; Pearson’s Chi-squared test.

**Table 2:** Multivariable logistic regression for delivery within 14 days.

Characteristic	OR (95% CI)	*p*-Value
Gestational age, weeks	1.164 (0.981–1.383)	0.083
log Cervical stiffness ^1^	0.907 (0.828–0.995)	0.039
Cervical length, mm	0.910 (0.876–0.946)	<0.001
Type of conception		
Natural	—	—
ART	2.082 (0.766–5.660)	0.151
Parity		
Nulliparous	—	—
Parous preterm delivery < 30 weeks	2.394 (0.780–7.341)	0.127
Parous preterm delivery 30–36 weeks	2.101 (0.344–12.825)	0.421
Parous term delivery ≥ 37 weeks	1.499 (0.641–3.502)	0.350
Cigarette smoker	4.378 (2.231–8.589)	<0.001

^1^ For cervical stiffness, the odds ratio corresponds to the average marginal effect because stiffness was modeled using restricted cubic splines. Abbreviations: ART, assisted reproduction techniques; OR, odds ratio; CI, confidence intervals.

**Table 3:** Adverse events within 7 days after measurement by causality assessment.

Adverse Event ^1^	Not Related	Possible	Probable	Definitely Related
Abdominal pain	2 (100.0%)	0 (0.0%)	0 (0.0%)	0 (0.0%)
Cervical bleeding	1 (14.3%)	4 (57.1%)	2 (28.6%)	0 (0.0%)
Chorioamnionitis	1 (100.0%)	0 (0.0%)	0 (0.0%)	0 (0.0%)
Decreased fetal movements	1 (100.0%)	0 (0.0%)	0 (0.0%)	0 (0.0%)
Persistent uterine contractions	2 (100.0%)	0 (0.0%)	0 (0.0%)	0 (0.0%)
Placental abruption	2 (100.0%)	0 (0.0%)	0 (0.0%)	0 (0.0%)
Urinary tract infection	1 (100.0%)	0 (0.0%)	0 (0.0%)	0 (0.0%)
Uterine irritability	1 (100.0%)	0 (0.0%)	0 (0.0%)	0 (0.0%)
Vaginal and suprapubic pain	1 (100.0%)	0 (0.0%)	0 (0.0%)	0 (0.0%)
Vaginal bleeding	0 (0.0%)	1 (33.3%)	1 (33.3%)	1 (33.3%)
Vulvovaginal candidiasis	1 (100.0%)	0 (0.0%)	0 (0.0%)	0 (0.0%)

^1^ Percentages were calculated by row.

## Data Availability

The data presented in this study are available on reasonable request from the corresponding author. The data are not publicly available due to privacy and ethical restrictions.
